# L1CAM Binds ErbB Receptors through Ig-Like Domains Coupling Cell Adhesion and Neuregulin Signalling

**DOI:** 10.1371/journal.pone.0040674

**Published:** 2012-07-16

**Authors:** Emanuelle Donier, Jose Antonio Gomez-Sanchez, Carmen Grijota-Martinez, Jarmila Lakomá, Sigrid Baars, Luis Garcia-Alonso, Hugo Cabedo

**Affiliations:** 1 Instituto de Neurociencias de Alicante, Universidad Miguel Hernández-Spanish National Research Council, San Juan de Alicante, Spain; 2 Fundación de la Comunidad Valenciana para la Investigación en el Hospital General Universitario de Alicante, Alicante, Spain; Aix Marseille University, France

## Abstract

During nervous system development different cell-to-cell communication mechanisms operate in parallel guiding migrating neurons and growing axons to generate complex arrays of neural circuits. How such a system works in coordination is not well understood. Cross-regulatory interactions between different signalling pathways and redundancy between them can increase precision and fidelity of guidance systems. Immunoglobulin superfamily proteins of the NCAM and L1 families couple specific substrate recognition and cell adhesion with the activation of receptor tyrosine kinases. Thus it has been shown that L1CAM-mediated cell adhesion promotes the activation of the EGFR (erbB1) from Drosophila to humans. Here we explore the specificity of the molecular interaction between L1CAM and the erbB receptor family. We show that L1CAM binds physically erbB receptors in both heterologous systems and the mammalian developing brain. Different Ig-like domains located in the extracellular part of L1CAM can support this interaction. Interestingly, binding of L1CAM to erbB enhances its response to neuregulins. During development this may synergize with the activation of erbB receptors through L1CAM homophilic interactions, conferring diffusible neuregulins specificity for cells or axons that interact with the substrate through L1CAM.

## Introduction

Immunoglobulin superfamily proteins are key players in the developmental mechanisms of metazoans. Two of them, NCAM and L1CAM, are involved in the control of morphogenesis, axon growth and guidance, and synaptic plasticity; but they have also other functions in and outside the nervous system. L1CAM behaves as an adhesion molecule in cell-aggregation assays. However L1CAM is more than a specific glue and serves as well as an activator of intracellular signaling pathways [1], [2], [3], [4]. L1CAM couples the highly specific recognition interaction mediated by homophilic adhesion with the activation of the EGFR (also known as erbB1). Thus, it has been reported that human-L1CAM homophilic adhesion promotes human-EGFR activation in transfected Drosophila-Schneider S2 cells [3]. This activity requires both homophilic binding and the expression of EGFR in the same cell, suggesting it is mediated by *cis*-interactions. During Drosophila development, the function of L1CAM (Neuroglian) is mediated by the EGFR, as revealed by the rescue of Neuroglian loss-of-function phenotype by activated-EGFR [4] and the suppression of Neuroglian gain-of-function phenotype by the loss of EGFR activity [3].The specificity of L1CAM as an activator of EGFR signaling has been conserved during the 500 million of years of evolution that separate Drosophila from human [5], [6]. The interaction of L1CAM with distinct molecular partners and the domains involved in these interactions have been well established [7], [8]. In contrast, it has not been possible to find evidence of physical binding between L1CAM and the EGFR, what could reflect a low affinity in the interaction [3]. Here we show evidence for this binding. We found that L1CAM, through the Ig-like domains, physically interacts with erbB receptors in heterologous systems. We also show evidences of the in vivo interaction of L1CAM with erbB receptors in the developing brain. Furthermore, we found that the interaction between L1CAM and erbB proteins strongly enhance the response of these receptors to their ligand neuregulin. Together with previous reports, our results support the view that the L1CAM-erbB interaction is an ancestral evolutionary-conserved mechanism that modulates erbB signaling. We propose this mechanism serves to increase the specificity of the neuregulin/erbB-signaling pathway, enhancing its precision and robustness for the control of cell migration and axon guidance during nervous system development.

## Results and Discussion

### L1CAM Physically Interacts with erbB Receptors in Heterologous Systems

**Figure 1:**
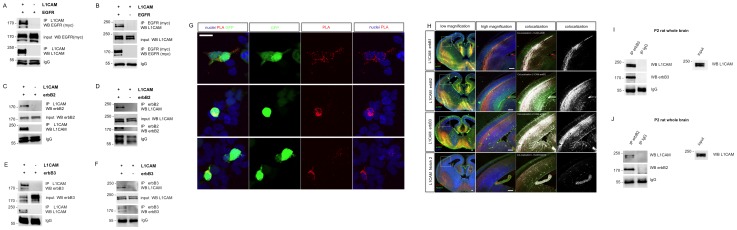
Physical interaction of L1CAM with erbB receptors. a ) L1CAM co-immunoprecipitates with erbB1 (EGFR): a pcDNA3 plasmid containing the cDNA encoding for human L1CAM and the pcDNA6A-EGFR construct were transiently co-transfected into the HEK293 cells. 48 h later cells were homogenized and L1CAM immunoprecipitated (IP). Immunoprecipitates were resolved by SDS-PAGE and blotted with anti-myc antibody to detect EGFR. As is shown, EGFR was pulled down only in L1CAM expressing cells. EGFR expression was similar in both extracts (input). Immunoblot with anti-L1CAM shows that this protein was correctly immunoprecipitated. **b**) Reverse co-immunoprecipitation. IP with anti-myc antibody pulls down L1CAM only in EGFR transfected cells. L1CAM expression was similar in both extracts (input). Immunoblot with anti-myc shows that the EGFR was correctly immunoprecipitated. **c**) L1CAM co-immunoprecipitates with erbB2: pcDNA3-L1CAM and the pcDNA3-erbB2 were transiently co-transfected into the HEK293 cells. Extracts were immunoprecipitated with the anti-L1CAM antibody. erbB2 was pulled down only in L1CAM expressing cells. erbB2 expression was similar in both extracts (input). Anti-L1CAM immunoblot shows that this protein was correctly immunoprecipitated. **d**) Reverse co-immunoprecipitation. IP with anti-erbB2 antibody pulls down L1CAM only in erbB2 transfected cells. L1CAM expression was similar in both extracts (input). Anti-erbB2 WB shows that erbB2 was correctly immunoprecipitated. **e**) erbB3 co-immunoprecipitates with L1CAM: pcDNA3-L1CAM and the pcDNA3-erbB3 were transiently co-transfected. Extracts were immunoprecipitated with the anti-L1CAM antibody. erbB3 was pulled down only in L1CAM expressing cells. erbB3 expression was similar in both extracts (input). Anti-L1CAM immunoblot shows that this protein was correctly immunoprecipitated. **f**) Reverse co-immunoprecipitation. IP with anti-erbB3 antibody pulls down L1CAM only in erbB3 transfected cells. L1CAM expression was similar in both extracts (input). Anti-myc WB shows that erbB3 was correctly immunoprecipitated. **g**) Proximity ligation assay showing L1CAM-erbB3 *in vivo* interaction. HEK293 cells were enforced to express L1CAM and erbB3. To identify the transfected cells, a plasmid encoding GFP was included. As is shown, only the transfected cells (green) were positive for the PLA signal (red). Note that the interaction signal can be detected in cells that are not in contact with other transfected cells, showing that the interaction between L1CAM and erbB3 is produced in *cis*. Scale bars represent 20 µm. **h**) L1CAM (red) co-localizes with EGFR (erbB1), erbB2 and erbB3 (green) in growing axons during brain development (at E14). Images at the right correspond to the co-localization channel (white). Co-localization is evident in cortical projections. Poor co-localization of L1-CAM was detected with Notch 2, used as a control for specificity. Co-localization was revealed with the ImageJ software and the Co-localization Finder plugin (for co-localization at P3 stage see the Figure S1). Images show coronal sections of E14 mouse brain incubated with the indicated antibodies and acquired at low magnification wide-field fluorescence (at left) or higher magnification under the confocal microscope. Scale bars correspond to 100 µm. **i**) L1CAM physically interacts with erbB3 *in vivo*. Whole brains of two days old rats were homogenized in RIPA buffer clarified by centrifugation and cross-linked with DTBP. Supernatants were immunoprecipitated with the anti-erbB3 antibody and blotted with anti-L1CAM. As a control of specificity an aliquot of the extract was immunoprecipitated with a non-specific anti-IgG. As shown, L1CAM was pulled down when immunoprecipitation was performed with the anti-erbB3 but not with the anti-IgG. Input shows that L1CAM is abundantly expressed in the P2 rat brains. IgG bands demonstrate a similar loading of immunoprecipitated proteins. This experiment was repeated 5 times. A representative experiment is shown. **j**) A similar result was obtained with the receptor erbB2.

It has been previously shown that human L1CAM-mediated homophilic cell interactions can activate the human EGFR tyrosine kynase activity in Drosophila S2 cells [3]. To explore if this is consequence of the interaction between both proteins in the plasma membrane, we checked whether L1CAM could physically bind the EGFR. To this aim, we subcloned the cDNA encoding for the human L1CAM (isoform 2) into the pcDNA3 mammalian expression vector (see Material and Methods section). Then, we co-transfected this construct and the pcDNA6A-EGFR (a vector that expresses the human EGFR with a myc epitope [9]) into the human embryonic kidney cells HEK293. Cells were trypsinized, harvested by centrifugation and lysed. Supernatants were incubated with anti-L1CAM monoclonal antibody prebound to protein A sepharose and immunoprecipated. Proteins were released and submitted to anti-myc western blot analysis. As shown in Figure 1a, myc immunoreactivity was pulled down from cells expressing L1CAM and EGFR, suggesting that both proteins physically interact when expressed in heterologous systems. The specificity of the immunoprecipitation (IP) was demonstrated by the absence of myc immunoreactivity pulled down from the cells that express the EGFR alone. To confirm this result, the reverse co-immunoprecipitation was performed. As is shown in Figure 1b, immunoprecipitation with anti-myc antibody was able to pull down L1CAM in cells that express EGFR. No immunoreactivity was detected when the EGFR was omitted. Together, these results demonstrate that L1CAM physically interacts with the EGFR in HEK293 cells.

EGFR belongs to the family of erbB receptors [10]. Four types of erbB receptors (erbB1-erbB4) that recognize different ligands have been described [11]. EGFR binds the epidermal growth factor (EGF), while erbB3 and erbB4 recognize members of the neuregulin family of proteins. Neuregulin binding to the erbB3 and erbB4 receptors induces heterodimerization with erbB2 receptor, which has a strong tyrosine kinase activity (but does not interact with known ligands). To explore whether L1CAM can also interact with other members of the mammalian erbB tyrosine kinase receptor family we co-transfected pcDNA3-L1CAM and pcDNA3-erbB2 into HEK293 cells. Cells were harvested, lysed and immunoprecipated with the anti-L1CAM antibody as described previously. As it is shown in Figure 1c, erbB2 receptor co-immunoprecipitated with L1CAM. The specificity of the assay was demonstrated by the absence of immunoreactivity immunoprecipitated from cells that express only erbB2 but not L1CAM. As before, to verify the interaction, the reverse experiment was performed. As is shown in Figure 1d, immunoprecipitation of erbB2 pulled down L1CAM, showing that L1CAM and erbB2 receptor are physically bound when expressed in HEK293 cells. No immunoreactivity was detected when erbB2 was omitted. A similar approach was used with the erbB3 receptor. As shown, L1CAM was also able to physically interact with erbB3 in HEK293 cells (Figure 1e and 1f). Thus far, our data show that L1CAM physically interacts with different members of the erbB family of tyrosine kinase receptors when expressed in vertebrate heterologous systems.

### In vivo Interaction of L1CAM and erbB3

Having determined that L1CAM can interact with different erbB receptors by co-IP assays, we decided to study whether the interaction occurs in intact cells in vivo by using the proximity ligation assay (PLA, see methods) [12]. To this aim, HEK293 cells were co-transfected with similar amounts of pcDNA-L1CAM and pcDNA-erbB3 plasmids. A smaller amount (1∶10) of a plasmid encoding for the green fluorescent protein was added to allow the identification of transfected cells. The interaction of L1CAM and erbB3 *in vivo* was determined using specific antibodies for each protein raised in different species and the “Duolink in situ” technology. As is shown in Figure 1g, a strong interaction signal (red dots) was observed only in transfected cells (GFP+) but not in non-transfected ones (GFP-). Thus far, our results demonstrated that L1CAM interacts with erbB3 in intact cells when expressed in heterlogous systems. PLA results also support a cis-interaction between L1CAM and erbB3, as we found strong signal in isolated cells where the L1CAM homophilic binding in trans is not possible.

L1CAM is expressed in different mammalian tissues where it is involved in many biological and pathological processes. One of these tissues is the nervous system [7], where L1CAM is pivotal for axon guidance and axon-glia interactions. Interestingly, neuregulin receptors are also highly expressed in the nervous system [13] being central for many aspects of its development [14]. Based on this we decided to explore whether L1CAM and neuregulin receptors physically interact in the nervous system *in vivo*. First we explored if L1CAM and erbB receptors are co-expressed. As shown in Figure 1 h, L1CAM strongly co-localizes with the EGFR in the growing axons of the developing mammalian brain, whereas much less co-localization was found with the non-related protein Notch 2. Interestingly, there is also a high degree of L1CAM co-localization with erbB2 and erbB3 receptors (Figure 1 h). The co-localization was also observed in the corpus callosum of P3 mice (Figure S1). Our previous data in heterologous systems and the high degree of colocalization strongly suggested the possibility of the *in vivo* interaction between L1CAM and the members of the erbB family of proteins. To check this hypothesis, brain extracts from P2 rats were immunoprecipitated with anti-erbB3 antibody and immunoblotted with the monoclonal anti-L1CAM antibody. To stabilize the interaction before IP we used DTBP, a cleavable, bifunctional, imidoester crosslinker (see Material and Methods). As is shown in Figure 1i, endogenously expressed L1CAM co-immunoprecipitates with erbB3 suggesting that both proteins physically interact in the developing brain. L1CAM co-immunoprecipitated as well with the erbB2 receptor from brain extracts (Fig 1j). However, we couldn’t detect L1CAM-EGFR co-immunoprecipitation (not shown), possibly reflecting a regulated protein-protein interaction. Nevertheless, it could be also consequence of technical problems related with the affinity of the interaction and/or the quality of the antibodies used for these studies. To confirm our observations, we explored the *in vivo* interaction of L1CAM and erbB3 by the PLA. As is shown in Figure S2, protein-protein interaction signal was observed in a subpopulation of cortical neurons at E14. These cells may correspond to previously identified L1CAM positive neurons in the marginal zone of the mouse developing brain. [15]. We couldn’t detect interaction in the growing axons possibly because the level of interacting proteins in these structures is below the limit of the PLA.

In summary our results show that L1CAM co-localizes and physically interacts with different members of the erbB family of proteins in the developing mammalian brain.

### The Ig-like Domains but not the Fibronectin Type III Repeats Determine L1CAM Interaction with erbB3

**Figure 2:**
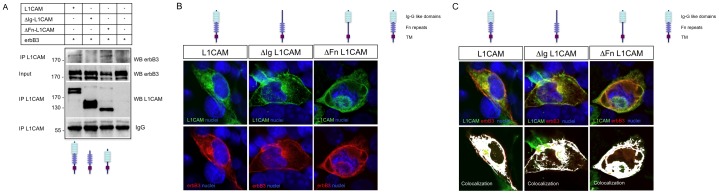
The Ig-like domains but not the fibronectin repeats of L1CAM mediate the physical interaction with erbB receptors. a) Ablation of Ig-like domains abrogates L1CAM interaction with erbB3: HEK293 cells were transfected with pcDNA3-erbB3 and the different truncated forms of L1CAM. Cell extracts were immunoprecipitated with anti-L1CAM antibody and blotted against erbB3. As is shown, erbB3 was pulled down when co-expressed with the full length and ΔFn-L1CAM constructs, but not when was co-expressed with the ΔIg-L1CAM construct. As expected, anti-L1CAM antibody does not immunoprecipate cells transfected with pcDNA3-erbB3 exclusively. Input lanes demonstrate the expression of erbB3 in the extracts. An aliquot of the immunoprecipitate was probed with anti-L1CAM to verify the adequate immunoprecipitation of the truncated proteins. IgG bands show that a similar amount of immunoprecipitate was loaded. This experiment was repeated three times. A representative experiment is shown. b) To rule out sorting problems that could explain the absence of co-IP, L1CAM and deleted constructs were co-transfected with erbB3 in COS-7 cells. As is shown, the distribution of L1CAM, ΔIg-L1CAM and ΔFn-L1CAM is similar when transfected into HEK293 cells, being detectable in the plasma membrane. c) The co-localization of the deleted constructs and full length L1CAM with erbB3 was nearly complete, ruling out sorting defects for the mutant proteins. L1CAM was detected with the anti-L1CAM monoclonal antibody (green) and erbB3 with a polyclonal antibody (red). Nuclei were counterstained with the Hoechst stain (blue). Co-localization (white) was revealed with the ImageJ software and the Co-localization Finder plugin.

The N-terminal domain of L1CAM consists of the Ig domains 1–6 followed by the fibronectin type III repeats 1–5. It has been shown that L1CAM specifically interacts with other partner proteins through different sequences of this extracellular domain [7], [8]. On this basis we decided to explore if the interaction of L1CAM with erbB receptors is mediated by some specific sequence in this part of the protein. First we removed the sequence encoding for the six Ig-like domains in the pcDNA3-L1CAM construct. The resulting deleted protein will be referred as ΔIg-L1CAM. We used a similar approach to remove the fibronectin type III repeats 1–5 (referred as ΔFn-L1CAM). Then we explored if the interaction with erbB receptors is preserved in these mutants. To this aim we co-transfected each of these constructs with erbB3 into HEK293 cells, and then performed co-immunoprecipitation assays as described previously. As it is shown in Figure 2a, truncated proteins of the expected molecular size can be immunoprecipitated from transfected cells (lower panel). Interestingly, whereas the ΔFn-L1CAM truncated protein retains its capacity to interact with erbB3, the elimination of the six Ig-like domains from the extracellular region (ΔIg-L1CAM) completely abrogates the capacity of L1CAM to interact with the erbB3 receptor. To rule out that deletions in L1CAM could produce sorting defects that prevent co-localization with erbB3, cells were transiently transfected with the L1CAM constructs and the pcDNA3-erbB3 vector. The expression and subcellular localization of L1CAM constructs and erbB3 was followed by immunofluorescence. As is shown in Figure 2b and c, both ΔIg-L1CAM and ΔFn-L1CAM truncated proteins were normally expressed, and co-localized almost perfectly with the erbB3 receptor, ruling out a sorting defect. Taken together our results indicate that some sequence in the Ig-like domain region of L1CAM mediates the interaction with erbB receptors.

### Different Ig-like Domains Support the Interaction of L1CAM with erbB3

**Figure 3:**
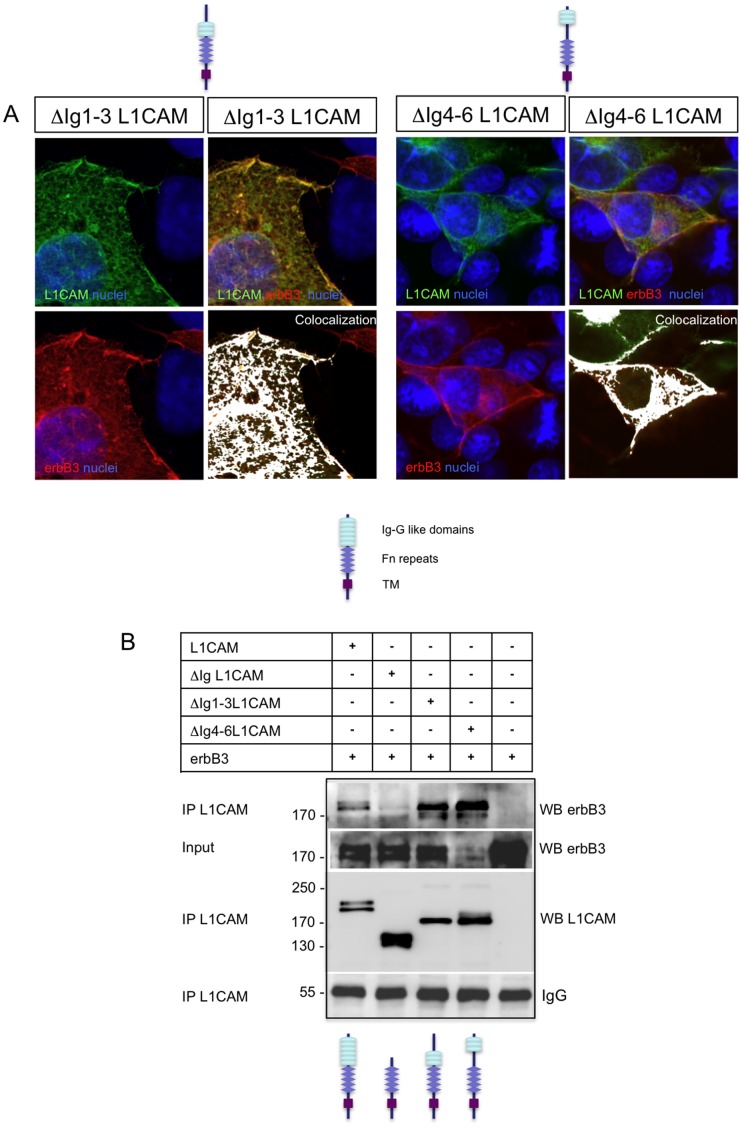
Different Ig-like domains can support L1CAM physical interaction with erbB receptors. **a**) Truncated proteins ΔIg1-3L1CAM and ΔIg4-6L1CAM are normally expressed and distributed when transfected into COS-7 cells. High magnification confocal images of cells transiently transfected with the indicated constructs are shown. L1CAM was detected with an anti-L1CAM monoclonal antibody (green) and erbB3 with a polyclonal antibody (red). Nuclei were counterstained with the Hoechst stain (blue). As is shown both deletion mutants of L1CAM co-localize with erbB3 (white). **b**) Ablation of Ig-like domains 1 to 3 or 4 to 6 does not abrogate L1CAM interaction with erbB3: HEK293 cells were transfected with pcDNA3-erbB3 and the different truncated forms of L1CAM. Cell extracts were immunoprecipitated with anti-L1CAM antibody and blotted against erbB3. As shown, erbB3 was pulled down when co-expressed with ΔIg1-3L1CAM, ΔIg4-6L1CAM and full-length constructs but not when co-expressed with the ΔIg-L1CAM construct. As expected, anti-L1CAM antibody does not immunoprecipitate erbB3 in cells transfected with pcDNA3-erbB3 exclusively. Input lanes demonstrate the expression of erbB3 in the extracts. An aliquot of the immunoprecipitated was probed with anti-L1CAM to verify the adequate expression and immunoprecipitation of the truncated proteins. IgG bands show that a similar amount of immunoprecipitated was loaded. This experiment was repeated twice. A representative experiment is shown.

To further characterize the specific sequence responsible for the interaction between L1CAM and erbB receptors we adopted a stepwise strategy. We started removing the Ig-like domains 1 to 3 (construct ΔIg1-3-L1CAM) and then the 4 to 6 in a different construct (ΔIg4-6-L1CAM). We first explored the expression of these proteins by immunofluorescence. As shown in Figure 3a, both truncated proteins are normally expressed and co-localize with erbB3. Then we co-transfected these truncated proteins, the full length L1CAM and the ΔIg-L1CAM construct, with erbB3 into HEK293 cells. Cells were lysed and the extracts immunoprecipitated with anti-L1CAM monoclonal antibody. As shown in Figure 3b, erbB3 co-immunoprecipitates with the ΔIg1-3-L1CAM construct, suggesting that the sequence that mediates L1CAM binding with erbB receptors lies in this region. However, and to our surprise, erbB3 was also able to co-immunoprecipitate with the ΔIg4-6L1CAM truncated protein. As expected, erbB3 did not co-immunoprecipitate with the construct that lacks the whole Ig-like region (ΔIg-L1CAM). Thus far our results show that different Ig-like domains of L1CAM can support the interaction with erbB receptors. Given the non-quantitative nature of our assay, we cannot rule out the existence of distinct Ig-like domain sequences with different affinities for erbB receptors. Interestingly the presence of erbB binding activity in the different Ig-like domains of L1CAM is consistent with the reported capacity of the vertebrate protein to activate Drosophila EGFR, as the Ig-like domains are well preserved between L1CAM and Neuroglian [5], [6]. This strongly suggests that the specificity of the physical interaction may reside in some general feature of the Ig-like domains and sheds light on why this may also happen for NCAM-type proteins, which have as well Ig-like domains [5]. Such a generic mechanism of interaction may be the reason why this type of erbB receptor control is strongly conserved during evolution.

### L1CAM-erbB Interaction Modulates Neuregulin Receptor Activation

**Figure 4:**
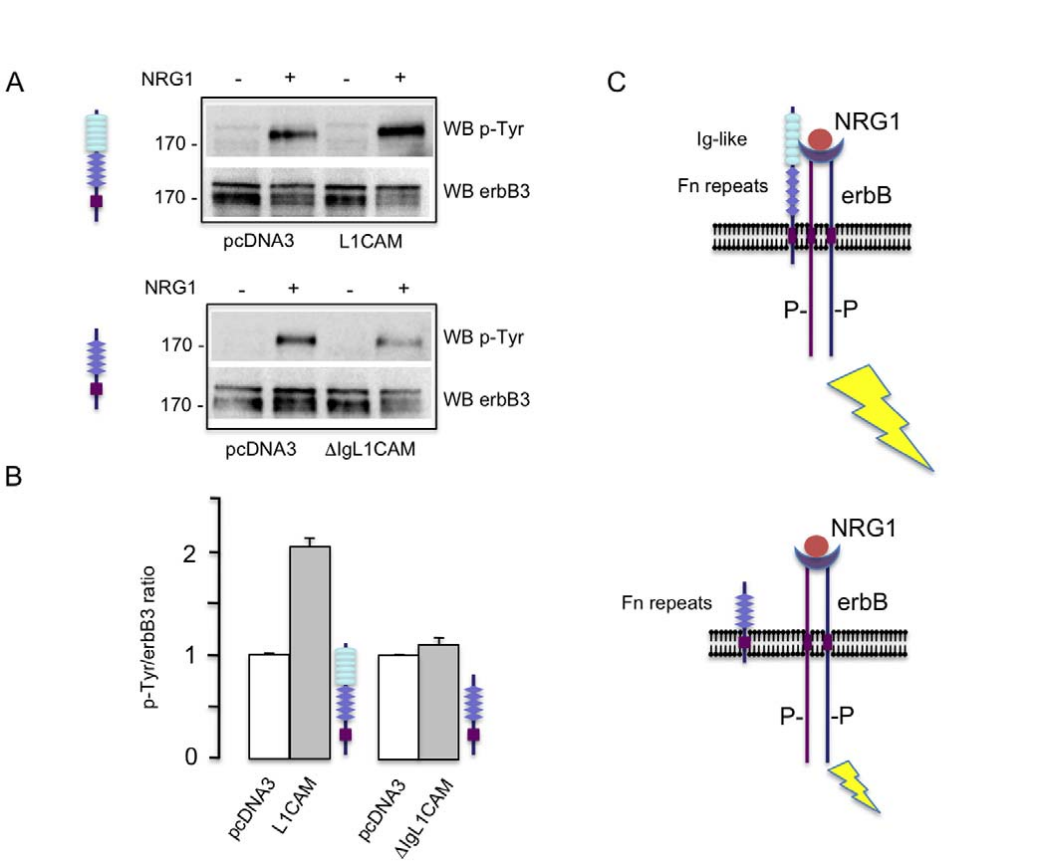
L1CAM-erbB interaction enhances neuregulin induced phosphorylation of erbB3. a ) Upper panel: MCF-7 cells were transiently transfected with pcDNA3-L1CAM or pcDNA3 empty vector. 24 h later, cells were serum starved and stimulated with recombinant NRG1 (50 nM) for 15 min. Then, cells were harvested and lysed. Extracts were submitted to SDS-PAGE and blotted with anti-p-Tyr monoclonal antibody or anti-erbB3 polyclonal antibody. This experiment was repeated three times. A representative experiment is shown. Lower panel: the same approach was used in cells transfected with the ΔIg-L1CAM truncated construct. This experiment was repeated twice. One of them is shown. **b**) Quantification of western blots by densitometry. The normalized amount of phosphorylated 180 kDa band is increased in cells that express the full length but not the truncated ΔIg-L1CAM protein, suggesting that the physical interaction of L1CAM and erbB3 is needed for the enhancing effect on neuregulin receptor activation. Bars represent standard errors **c**) Proposed model: the interaction with L1CAM sensitizes erbB receptors to the activation by neuregulins. Removing the Ig-like rich region of L1CAM prevents the interaction and avoids receptor sensitization. For simplicity, only *cis*-interactions are depicted in the model.

Upon neuregulin binding erbB intracellular domain becomes autophosphorylated and recruits cytosolic proteins that activate intracellular signalling pathways [16], [17]. Although initially identified as a cell adhesion molecule, L1CAM has been shown to be pivotal for cell-to-cell signalling in different biological contexts [8], [18], [19], [20]. As it has been introduced previously, human L1CAM-mediated homophilic cell interactions activate the human EGFR tyrosine kynase activity in Drosophila S2 cells [3]. With this in mind, we reasoned that the physical interaction with L1CAM could modulate the sensitivity of erbB receptors to activation by ligands. To explore this hypothesis we used the cell line MCF-7, a breast cancer cell that expresses endogenously erbB2 and erbB3 receptors [21]. First we transiently transfected MCF-7 cells with the cDNA encoding for the full length L1CAM. The enforced expression of L1CAM produces no changes in the expression of endogenous erbB3 (Fig S3). Then cells were challenged with neuregulin. As a control we transfected MCF-7 cells with the empty vector (pcDNA3). Cells were harvested and the activation status of erbB receptors explored. In MCF-7 cells this can be done in immunoblots by determining the amount of anti-phosphotyrosine immunoreactivity in the ≈ 180 kDa region [9], [22], [23], [24]. As shown in Figure 4a, enforced L1CAM expression increased the phosphorylation in tyrosines of the 180 kDa band, suggesting that physical interaction between L1CAM and erbB2/erbB3 sensitizes the receptor complex to the activation by neuregulins. To test this hypothesis we transfected MCF-7 cells with ΔIg-L1CAM, the truncated protein that, as shown in the previous points, is unable to interact with erbB3. As it is shown in Figure 4b, the ablation of the Ig-like domain region completely abrogates the capacity of L1CAM to sensitize the erbB2/erbB3 complex to neuregulins. Taken together, our data shows that the physical interaction of L1CAM with neuregulin receptors modulates the response of erbB proteins to ligands and suggests a role of the interaction in the regulation of the intracellular signalling cascades elicited by these proteins. Note that our experiments are performed in cultures with a low degree of confluence, where the *cis*-interactions predominate over *trans*-interactions. Also, the interaction of L1CAM with erbB3 can occur in “isolated” cells where the L1CAM mediated homophilic cell adhesion is not possible (Figure 1g). Therefore, our results strongly suggest that it is the interaction of L1CAM and erbB in *cis* what sensitizes these receptors for neuregulin signalling. However, we do not rule out that L1CAM homophilic binding could also modulate the *cis*-interactions of L1CAM and erbB receptors in confluent cultures.

Development of complex tissues like the nervous system relies on the deployment of many different cell-cell communication processes in parallel. It is likely that mechanisms coupling specific substrate recognition and adhesion with signalling by attractants and repellents help to increase the precision and robustness of cell migration, axon guidance, target recognition and synaptogenesis. Indeed, this may be the reason why the L1CAM Ig-like domain interaction with erbB receptors is a mechanism preserved during more than half billion years of metazoan evolution.

## Material and Methods

### Materials

Pfu turbo DNA polymerase and BL21 codon plus *E. coli* strain were from Stratagene. HRP-conjugated anti-rabbit IgG and anti-mouse IgG secondary antibodies, monoclonal antiphosphotyrosine (clone PT-66) were obtained from SIGMA. Anti-L1CAM monoclonal antibody (ab24345) and anti-IgG polyclonal antibody (ab27478) were from Abcam. Anti-erbB3 (C-17; sc-285) polyclonal antibody was from Santa Cruz Biotechnology. Anti-erbB2 (29D8) polyclonal antibody and anti-EGFR (C74B9) were from Cell Signalling. ECL+plus was from Amersham biosciences. Lipofectamine 2000 was obtained from Invitrogen. The pcDNA3-erbB2 and pcDNA3-erbB3 vectors were kindly provided by Professor Yossef Yarden (The Weizmann Institute of Science, Rehovot, Israel). pcDNA6A-EGFR was obtained from MC Hung (University of Texas M.D. Anderson Cancer Center, Houston, USA) [9].

#### Subcloning of L1CAM and production of deletions

The cDNA encoding for the human L1CAM (isoform 2) was subcloned into the pcDNA3 vector. This construct was used as a template to obtain the pcDNA3-L1CAM ΔIg and ΔFn constructs and variants by standard molecular biology techniques.

#### Cell lines, culture and transfections

HEK293 and MCF-7 cells (both from ATCC-LGC) were cultured in Dulbecco’s modified Eagle’s medium (DMEM) containing 10% of foetal bovine serum. Cells were plated on 2 cm^2^ wells at 250.000 cells/well. Twenty hours later, cells were transfected with 1 µg of plasmid DNA using lipofectamine 2000 following the manufacturer recommendations. MCF-7 cells were cultured in DMEM containing 10% of foetal bovine serum.

#### Tyrosine phosphorylation assay

Neuregulin-induced tyrosine phosphorylation of erbB receptors was carried out as described previously [9], [22], [23], [24]. Briefly, MCF-7 cells were grown until ≥80% confluence in 24–well plates. Thereafter, cells were serum-starved for 2–5 h and incubated with recombinant neuregulin for 15 minutes at room temperature as indicated. Medium was removed and cells were harvested and homogenized in RIPA buffer. Whole cell extracts were heat denatured, separated by SDS-PAGE, and analyzed by immunoblotting with the monoclonal anti-phosphotyrosine antibody (1∶1,000). Purification of the recombinant neuregulin from E. coli was performed as described elsewhere [25]. Protein concentration was calculated with the method of Bradford [26] or BCA (Pierce).

#### Immunofluorescence

Cells were seeded on cover-slips and transfected with the indicated vectors. Primary antibody (anti-L1CAM at 1∶1000) was diluted in 1% goat serum and 0.3% Triton X-100 in PBS and incubated overnight at 4°C. Cover-slips were then washed with PBS, and detection was performed using the fluorescent secondary antibody anti-mouse AlexaFluor 488 (Invitrogen) at 1∶700 dilution for 1 h. Nuclei were counterstained with bisbenzimide (Hoechst nuclear stain) in PBS. Samples were mounted in Fluoromount G (Southern Biotechnology Associates). For tissue immunofluorescence we obtained 30–50 µm floating-sections from embryonic and P3 mouse brains. Primary antibodies were used at dilutions: mouse anti-L1CAM, 1∶300; rabbit anti-EGFR, 1∶100; rabbit anti-erbB2, 1∶100 and rabbit anti-erbB3, 1∶100. We used Cy2- and Cy3-coupled secondary antibodies from Jackson ImmunoResearch. DAPI was used to stain nuclei in the brain sections. Images were obtained using a confocal ultraspectral microscope (Leica TCS SP2).

#### Co-immunoprecipitation

HEK293 cells were transfected with Lipofectamine 2000 according to the manufacturer’s instructions. 24 h post-transfection, cells were lysed (lysis buffer: 50 mM Tris, 150 mM NaCl, 2 mM EDTA, protease inhibitor cocktail (Complete Mini, Roche), 0.5% Triton X-100) and incubated 1 h on ice. Cell lysate was centrifuged 10 min at 4°C, and an aliquot of the supernatant was kept aside on ice (“input”). Protein A-Sepharose beads (GE Healthcare) were loaded with the primary antibody for 1 h at room temperature and washed 3 times with PBS. Cell lysate supernatant was mixed with antibody-loaded beads, and incubated 3 h on ice, with mild shaking. Beads were washed 4 times with ice-cold PBS, resuspended in SDS sample buffer, boiled 5 min, and loaded onto a 7% acrylamide gel. The proteins were transferred to nitrocellulose membrane (Protran, Whatman GmbH). The membrane was blocked with 5% BSA in TBS containing 0.1% Tween and incubated with the indicated antibody in blocking buffer overnight at 4°C. The membrane was then washed three times with TBS containing 0.1% Tween, and the secondary antibody (horseradish peroxidase-conjugated) was applied at 1∶2000 dilution in TBS containing 0.1% Tween for 2 h at room temperature. Immunoreactivity was detected using ECL Plus detection reagent (GE Healthcare). A similar protocol was used with the brains removed from euthanized P2 rats. In this case, the interaction of the proteins was stabilized with the cleavable, bifunctional, imidoester crosslinker DTBP (3 mM, 45 min) befote IP.

#### Proximity ligation assay (PLA)

The in situ PLA method allows determine the subcellular localization of protein–protein interactions. Oligonucleotides attached to antibodies against two target proteins guide the formation of circular DNA strands when bound in close proximity. The DNA circles serve as templates for localized rolling-circle amplification, allowing individual interacting pairs of protein molecules to be visualized [12]. HEK293 cells transfected with the pcDNA3-L1CAM, pcDNA3-erbB3 and pEGFP were seeded on coverslips, fixed and processed with the Duolink In Situ kit (OLINK Bioscience) as recommended by manufacturer. Duo-link was used with the primary antibodies mouse anti-L1CAM and rabbit anti-erbB3. Secondary anti-mouse and anti-rabbit antibodies attached to oligonucleotides were used as proximity probes. After hybridization, ligation and amplification, a detection solution containing a fluorescent probe was added. Fluorescent spots were then visualized by confocal microscopy. Negative controls were non-transfected cells. The same approach was used with E14 mouse brain free-floating sections.

#### Ethics statement

To avoid suffering animals were profoundly anesthetized before euthanasia. All animal work has been conducted according to EU guidelines and with protocols approved by the “Comité de Bioética y Bioseguridad del Instituto de Neurociencias de Alicante UMH-CSIC” (http://in.umh.es/).

## Supporting Information

Figure S1 **Expression of EGFR erbB2 and erbB3 receptors (in green) and L1CAM (in red) in P3 mouse brain.** L1CAM co-localizes with EGFR, erbB2 and erbB3 in the Corpus Callosum of P3 mouse brain. Low magnification is shown in left panels and high magnification in middle panels. Images at right correspond to the co-localization channel (white). Co-localization is evident in the callosal tract at P3. Poor co-localization of L1CAM with Notch 2 can be observed. Co-localization was revealed with ImageJ software and the Co-localization Finder plugin.
(PDF)

Figure S2 **PLA performed on free-floating sections from E14 mouse brain confirmed** ***in vivo*** **the interaction of L1CAM with erbB3.** A group of neurons in the cortex gave a strong PLA signal (red). Nuclei were counterstained with the Hoechst staining. These neurons were tentatively identified as “pioneer neurons” by the expression of L1CAM and the topographical localization in the E14 cortex (see text). However, we couldn’t detect interaction signal in the axons. Bar represent 40 µm.
(PDF)

Figure S3 **a) L1CAM enforced expression doesn’t change the expression levels of the endogenous erbB3 or erbB2 expression in MCF-7 cells.** MCF-7 cells were transiently transfected with the pcDNA3-L1CAM expression vector. 24 h later, cells were immunostained for L1CAM (green) and erbB3 (red). Nuclei were counterstained with the Hoechst nuclear stain. As is shown, no differences in endogenous erbB3 expression can observed in those cells that have been transfected with L1CAM. **b)** The same result was obtained for the ΔIg-L1CAM construct. **c)** Levels of erbB2 were also non-changed by the expression of L1CAM.
(PDF)
