# Supplementary figures and images for: L1CAM Binds ErbB Receptors through Ig-Like Domains Coupling Cell Adhesion and Neuregulin Signalling
Source: PLoS One. 2012 Jul 16;7(7):e40674. doi: 10.1371/journal.pone.0040674 (PMC3398014; doi:10.1371/journal.pone.0040674)

Supplementary Figure 1

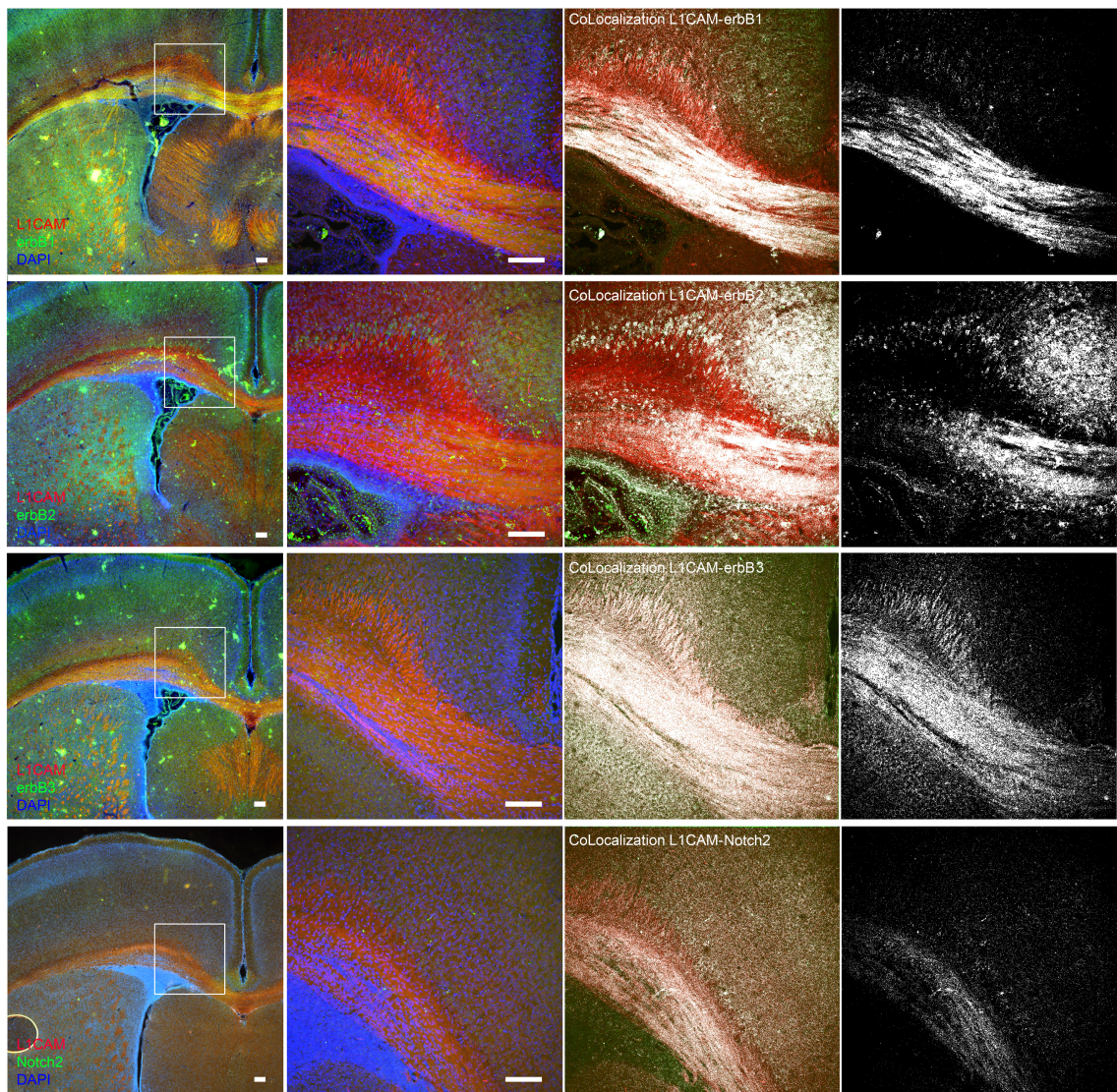

Supplement: Figure S1 — Expression of EGFR erbB2 and erbB3 receptors (in green) and L1CAM (in red) in P3 mouse brain. L1CAM co-localizes with EGFR, erbB2 and erbB3 in the Corpus Callosum of P3 mouse brain. Low magnification is shown in left panels and high magnification in middle panels. Images at right correspond to the co-localization channel (white). Co-localization is evident in the callosal tract at P3. Poor co-localization of L1CAM with Notch 2 can be observed. Co-localization was revealed with ImageJ software and the Co-localization Finder plugin. (PDF) [file pone.0040674.s001.pdf]

Supplementary Figure 2

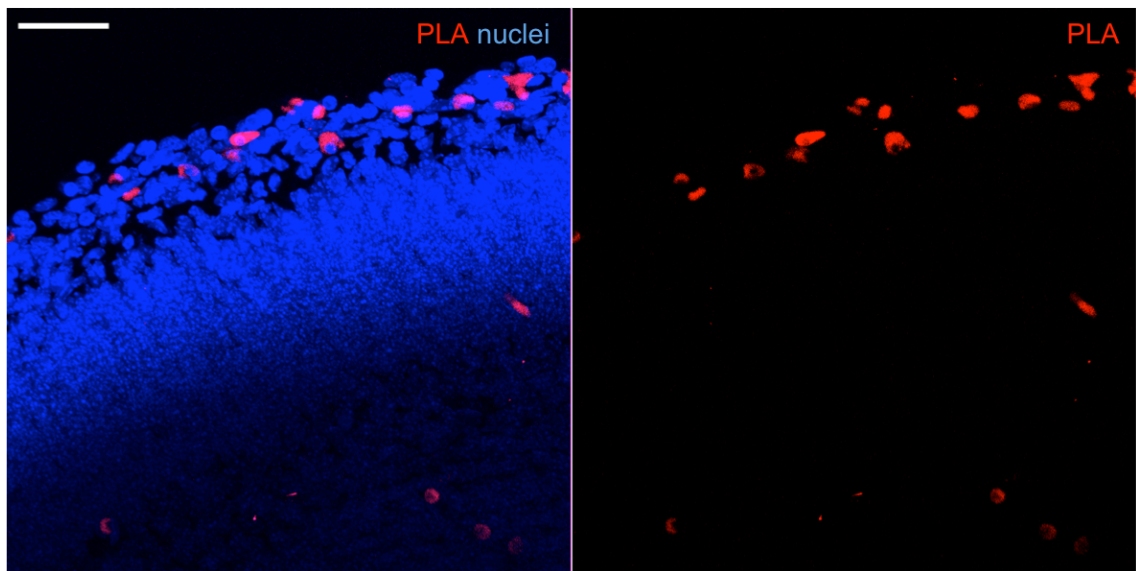

Supplement: Figure S2 — PLA performed on free-floating sections from E14 mouse brain confirmed in vivo the interaction of L1CAM with erbB3. A group of neurons in the cortex gave a strong PLA signal (red). Nuclei were counterstained with the Hoechst staining. These neurons were tentatively identified as “pioneer neurons” by the expression of L1CAM and the topographical localization in the E14 cortex (see text). However, we couldn’t detect interaction signal in the axons. Bar represent 40 µm. (PDF) [file pone.0040674.s002.pdf]

Supplementary Figure 3

A

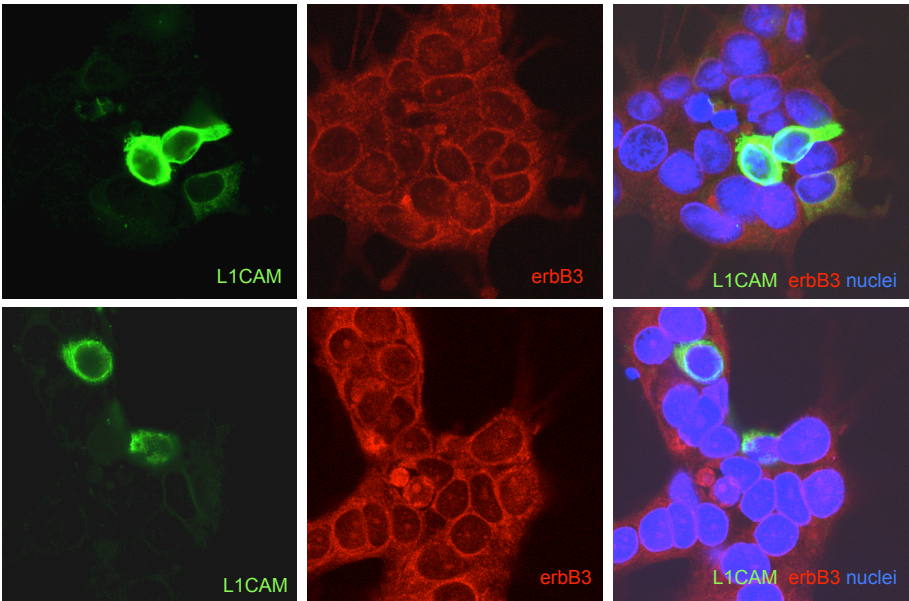

B

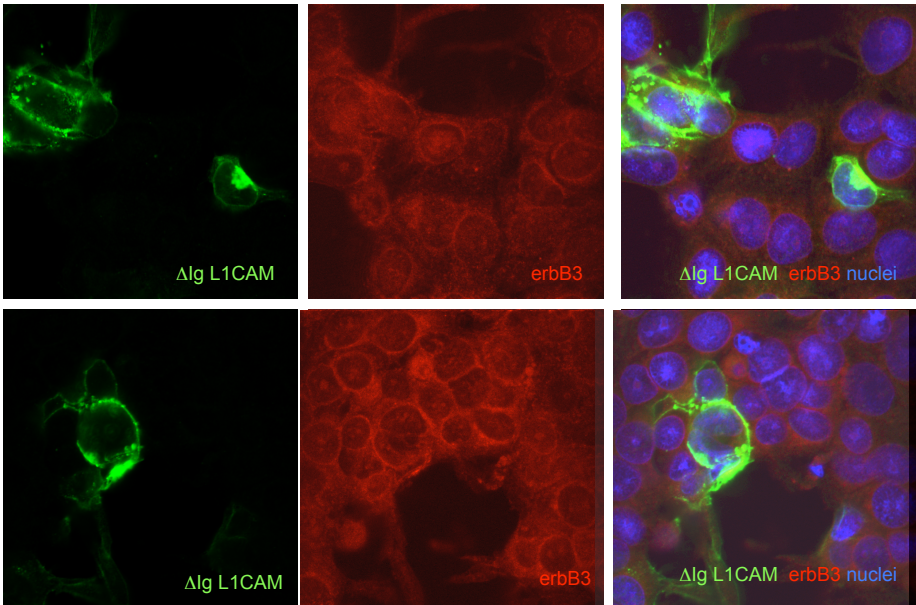

C

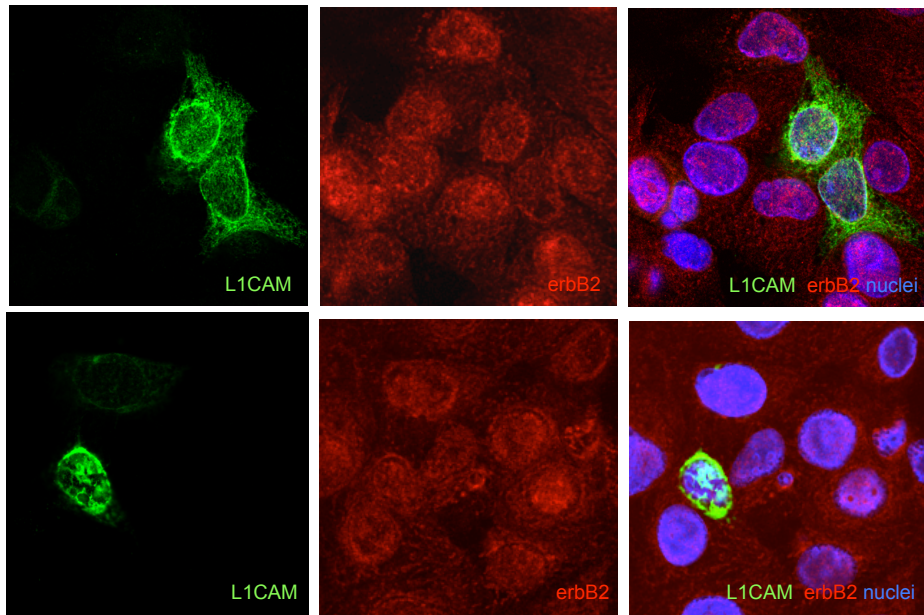

Supplement: Figure S3 — a) L1CAM enforced expression doesn’t change the expression levels of the endogenous erbB3 or erbB2 expression in MCF-7 cells. MCF-7 cells were transiently transfected with the pcDNA3-L1CAM expression vector. 24 h later, cells were immunostained for L1CAM (green) and erbB3 (red). Nuclei were counterstained with the Hoechst nuclear stain. As is shown, no differences in endogenous erbB3 expression can observed in those cells that have been transfected with L1CAM. b) The same result was obtained for the ΔIg-L1CAM construct. c) Levels of erbB2 were also non-changed by the expression of L1CAM. (PDF) [file pone.0040674.s003.pdf]
